# Mechanism of DNA Intercalation by Chloroquine Provides Insights into Toxicity

**DOI:** 10.3390/ijms25031410

**Published:** 2024-01-24

**Authors:** Joha Joshi, Micah J. McCauley, Michael Morse, Michael R. Muccio, Joseph G. Kanlong, Márcio S. Rocha, Ioulia Rouzina, Karin Musier-Forsyth, Mark C. Williams

**Affiliations:** 1 Department of Physics, Northeastern University, Boston, MA 02115, USA; j.joshi@northeastern.edu (J.J.); m.mccauley@northeastern.edu (M.J.M.); mi.morse@northeastern.edu (M.M.); 2 Department of Chemistry and Biochemistry, Center for RNA Biology, Ohio State University, Columbus, OH 43210, USA; muccio.20@buckeyemail.osu.edu (M.R.M.); kanlong.1@buckeyemail.osu.edu (J.G.K.); irouzina@gmail.com (I.R.); musier-forsyth.1@osu.edu (K.M.-F.); 3 Department of Physics, Universidade Federal de Viçosa, Viçosa CEP 36570-900, MG, Brazil; marcios.rocha@ufv.br

**Keywords:** chloroquine, intercalation, DNA binding, single molecule, optical tweezers, AFM, DNA melting, isothermal titration calorimetry

## Abstract

Chloroquine has been used as a potent antimalarial, anticancer drug, and prophylactic. While chloroquine is known to interact with DNA, the details of DNA–ligand interactions have remained unclear. Here we characterize chloroquine–double-stranded DNA binding with four complementary approaches, including optical tweezers, atomic force microscopy, duplex DNA melting measurements, and isothermal titration calorimetry. We show that chloroquine intercalates into double stranded DNA (dsDNA) with a *K_D_* ~ 200 µM, and this binding is entropically driven. We propose that chloroquine-induced dsDNA intercalation, which happens in the same concentration range as its observed toxic effects on cells, is responsible for the drug’s cytotoxicity.

## 1. Introduction

**Figure 1:**
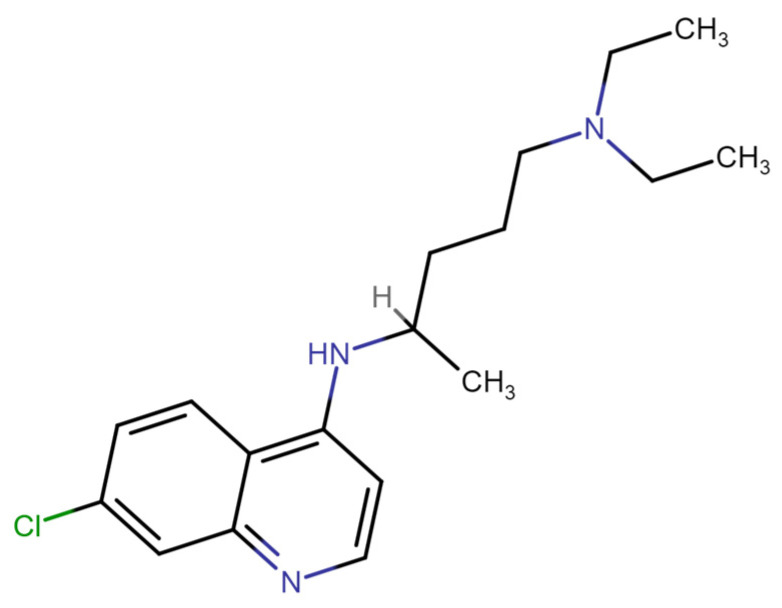
Structure of chloroquine, showing aromatic rings that may intercalate into DNA and a basic tail.

Chloroquine (also known as chloroquine phosphate) has been widely employed in both the treatment and prevention of malaria [1]. Chloroquine has also been used to treat other diseases, including rheumatoid arthritis and cancer [2]. It was also suggested as an effective antiviral during the COVID-19 pandemic, though the effectiveness of chloroquine in this role has not been effectively proven [3]. Furthermore, there are significant side effects to this treatment, as chloroquine active doses for COVID-19 treatment are higher than for antimalarial treatment, while its cytotoxicity for the antiviral application is more significant [4,5]. While most of chloroquine’s medicinal activities as a drug are related to its ability to penetrate cellular organelle membranes and to change their pH [6,7], leading to the alkalinization of parasite lysosomes [8] and binding to hemin [9], the molecular basis for chloroquine’s high cytotoxicity remains unclear. As shown in Figure 1, chloroquine consists of a pair of aromatic rings and a longer, unstructured tail, which is weakly positively charged at pH 7.5. Despite the interest in and the known toxicity of this compound, few studies have systematically probed DNA–chloroquine interactions since the 1940s, when this drug was first introduced and studied. It was established that chloroquine is a DNA intercalator, as binding increases DNA viscosity and rigidity while changing its spectral properties [10]. A recent study examined chloroquine–DNA intercalation with optical tweezers (OTs). However, the applied stretching forces were too low (<1.5 pN) to observe significant chloroquine intercalation under the conditions studied [11].

Here, we performed stretching experiments with our OT instrument under a much broader force and chloroquine concentration range. We find that chloroquine behaves as a classic double-stranded DNA (dsDNA) intercalator at high concentrations, increasing the contour and persistence length in a force-dependent manner. At low concentrations, we also observe an increase in DNA persistence length without DNA lengthening. To understand this behavior, we use the OT approach developed previously for other intercalators, including planar intercalators such as ethidium [3] and more complex structures [12,13,14,15] to fully characterize binding both in the presence and absence of DNA tension while also demonstrating binding using AFM. We find that chloroquine intercalates dsDNA at ~200–300 µM in physiological salt, extending the dsDNA duplex by ~0.29 nm/bound ligand. This intercalation is weakly salt-dependent and is entropically driven. We also show that saturated chloroquine intercalation increases the dsDNA melting temperature by ~15 °C. Intercalation stabilizes B-DNA; this can potentially interfere with dsDNA function in cells and lead to cytotoxicity in anti-COVID-19 treatment protocols.

## 2. Results

### 2.1. Force Extension of dsDNA + Chloroquine in Optical Tweezers

**Figure 2:**
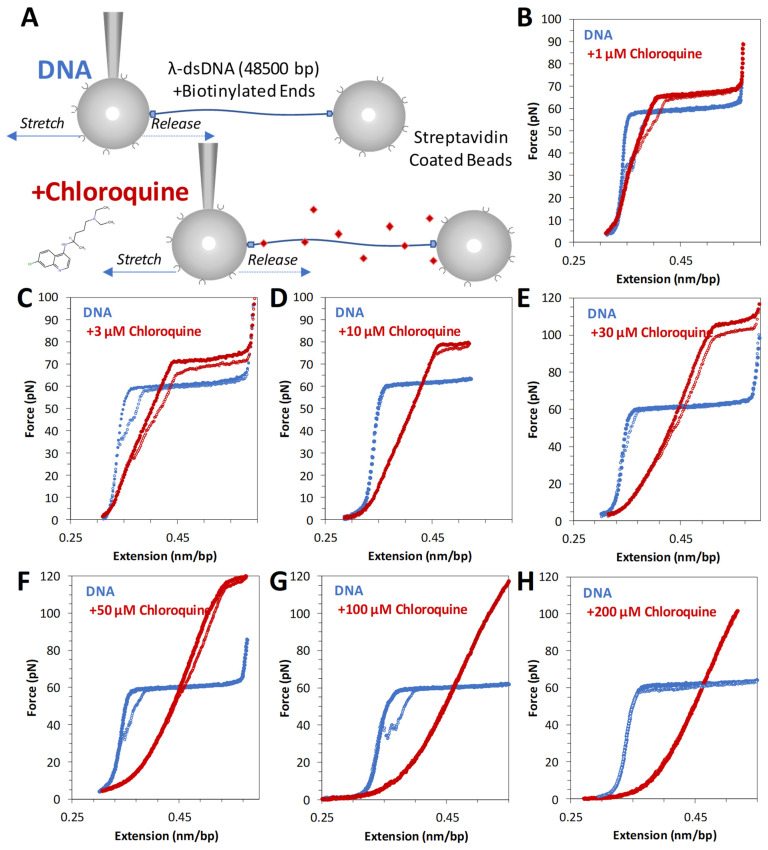
Force extension of λ DNA in the absence and presence of chloroquine (red diamonds). (**A**) Schematic of λ DNA tethering in optical tweezers experiment. Representative force extension and release data (blue solid circles and open circles, respectively) for λ DNA stretched in the presence of (**B**) 1 μM, (**C**) 3 μM, (**D**) 10 μM, (**E**) 30 μM, (**F**) 50 μM, (**G**) 100 μM, and (**H**) 200 μM chloroquine (red circles).

The schematic for stretching DNA in an OT assay is shown in Figure 2A. Tethered DNA is stretched and released as described in the Methods. Pulling dsDNA follows a well-studied trajectory. After initial enthalpic and entropic stretching regimes, described by the worm-like chain (WLC) polymer model (Equation (1) in Section 4), dsDNA abruptly increases in length. At an almost constant force of ~60 pN, lengthening signifies the cooperative transition into the ~1.7-fold longer DNA state [16]. Depending upon the solution and other experimental conditions, base pairing may or may not be lost, leading to the formation of single stranded DNA (ssDNA) or double-stranded S-form DNA. Under the conditions of these experiments, this transition is fully reversible upon the release of tension, suggesting a rapid transition between the two dsDNA forms, i.e., a B-to-S transition rather than dsDNA melting [17,18].

Titrating increasing concentrations of chloroquine from 1 µM through 50 µM (Figure 2B–F) reveals that the measured DNA length (the contour length) increases, as does the height of the overstretching transition force plateau. At 100 µM and above (Figure 2G,H), the overstretching plateau disappears entirely. Furthermore, these changes are fully reversible (i.e., extension and release data nearly overlap), indicating that the chloroquine-dsDNA force-induced binding and unbinding during the stretch–release cycle (~20 s) happen in equilibrium. At least 3 consecutive cycles were collected at each concentration to verify reproducibility and estimate uncertainty. Observed chloroquine–dsDNA stretching curves closely resemble the analogous curves for the ethidium–dsDNA complex, although at about ~100-fold lower ligand concentrations. We, therefore, analyze our chloroquine–dsDNA data with the previously developed approach [19].

### 2.2. Characterizing Chloroquine Binding to dsDNA

**Figure 3:**
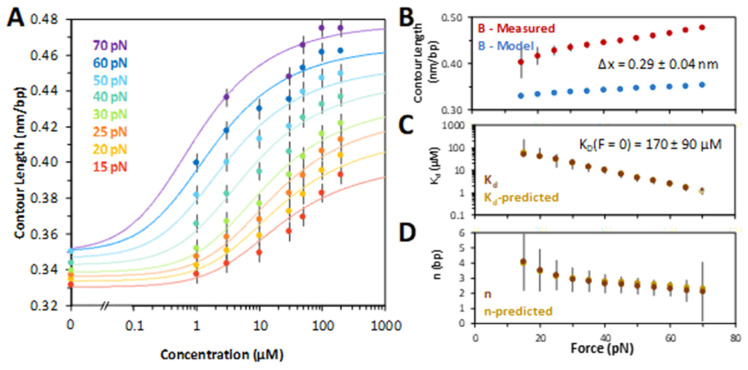
Chloroquine titration with DNA determines binding parameters. (**A**) Chloroquine titration with DNA (from Figure 2) reveals the change in DNA length versus ligand concentration across forces ranging from 15 pN (red) through 70 pN (purple), including the ligand-free length below overstretching (note the break ‘//’ in the *x* axis). Solid lines are χ^2^ minimized fits to the binding isotherms, as described in the Methods. (**B**) Values of the measured chloroquine induced DNA end to end (contour) length (red) compared to models of DNA elasticity (blue), as described in the text. High force (>60 pN) yields a change of Δ*x* = 0.29 ± 0.04 nm. (**C**) Fitted equilibrium dissociation constant (*K_D_*) and (**D**) binding site size (*N*) at each force, as described in the text. The equilibrium dissociation may be extrapolated to find a value in the absence of any external force of *K_D_*(*F* = 0) = 170 ± 90 μM (Equation (4), Section 4). The force and extension errors are standard errors from three DNA stretches. Uncertainties in *K_D_*, Δ*x*, and *N* are all deduced from values of χ^2^ + 1.

At fixed forces, the DNA extension was measured as a function of ligand concentration. The chloroquine–dsDNA titration curves are plotted in Figure 3A. Each titration curve was fit to a model of ligand–polymer binding (Equations (2) and (3) in Section 4), yielding the contour length of the chloroquine–dsDNA complex, its dissociation constant, *K_D_*, and the binding site size in base pairs, *N*, as a function of the stretching force F. Fitting the *K_D_*(*F*) dependence to the exponential (Equation (4) in Section 4), we obtained the dsDNA elongation per chloroquine intercalation event, Δ*x* = 0.29 ± 0.04 nm (Figure 3B). The extrapolated chloroquine dissociation constant in the absence of force, *K_D_*(*F* = 0), was determined to be 170 ± 90 µM (Figure 3C). This fitting procedure involves non-linear, iterative steps (Equations (2) and (3), Section 4), allowing us to determine all three parameters (Figure 3B–D). The dissociation constant and the binding site size (Figure 3C,D) both decrease with force, suggesting facilitation of intercalation by the stretching force. In the absence of force and at chloroquine saturation, the ligand intercalates every 5th base pair stack (*N* ~ 5 at low force).

### 2.3. Chloroquine Binding to ssDNA Is Weaker Than to B-DNA

**Figure 4:**
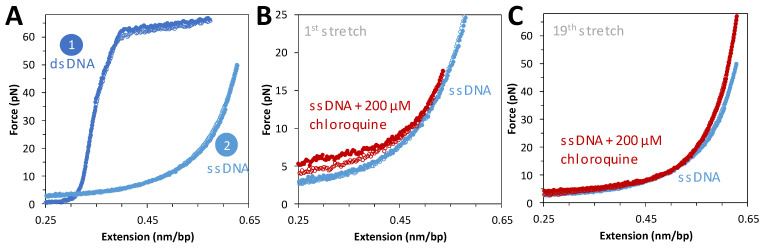
ssDNA stretching curves in the presence of 200 μM of chloroquine. (**A**) Representative stretch and release curves for 8.1 kbp dsDNA in dark blue ((**A**), 1). After returning to its original position, 20 μL of 5 M NaOH is added to the flow cell to melt the DNA. A representative ssDNA stretch curve is shown in light blue ((**A**), 2). (**B**) Representative curve for the first stretch of ssDNA in the absence (blue) and presence of 200 μM chloroquine at 20 nm step size, with total time for extension and release of ~10 s (red closed and open circles, respectively). (**C**) The same DNA molecule on its nineteenth stretch in the absence (blue) and presence (red) of chloroquine.

To complement force extension data on dsDNA, tethered DNA constructs were chemically denatured and then exposed to saturating concentrations of chloroquine. Figure 4A shows force extension data for the same molecule before and after denaturation. Flowing in a saturating concentration of chloroquine and repeating the cycle of extension and release reveals a slight increase in the measured force during extension (Figure 4B). This is attributed to the secondary structure (hairpin formation) formed when ssDNA is relaxed, which is modestly stabilized by chloroquine binding during introduction into the flow cell. After a few cycles, this stabilization disappeared (Figure 4C), with no other effects on the ssDNA force–extension curve. The secondary structures initially observed have been pulled out and do not reform under these conditions. This result suggests that at 200 μM chloroquine, when binding to dsDNA is almost saturated, no effect on ssDNA is observed. We conclude that chloroquine binding to ssDNA is much weaker than to dsDNA.

### 2.4. Chloroquine Intercalation Stabilizies B-DNA

**Figure 5:**
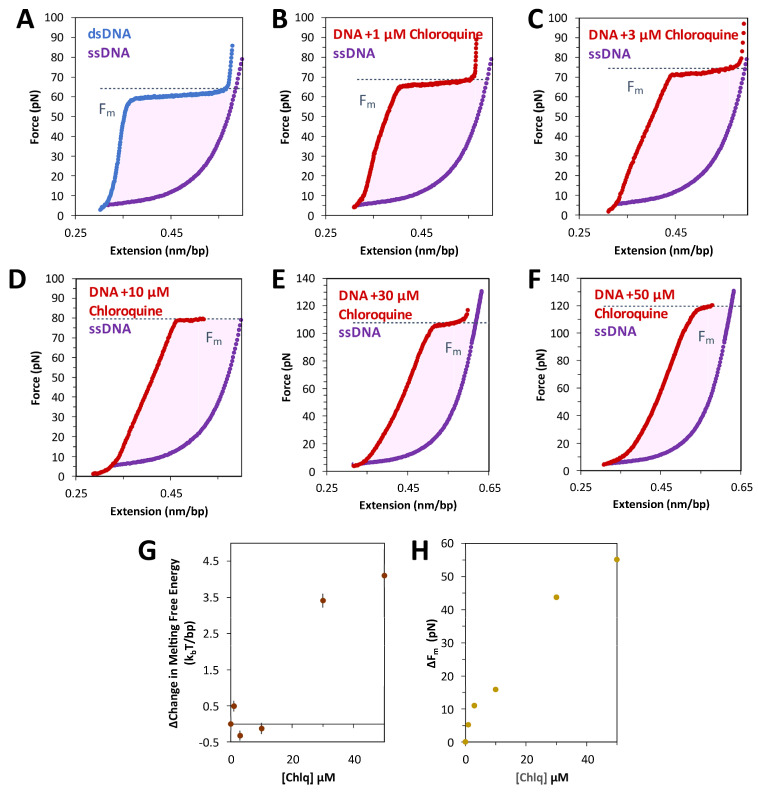
Free energy of DNA overstretching increases with chloroquine binding. The energy of converting dsDNA to ssDNA, or base melting, is shown as the shaded, integrated area between the force extension data for dsDNA (blue without chloroquine, red with chloroquine) and ssDNA (purple), below the critical melting force (*F_m_*, dotted line). This value may be found for each ligand concentration where melting is observed; in the absence of (**A**) and in the presence of (**B**) 1 µM, (**C**) 3 µM, (**D**) 10 µM, (**E**) 30 µM, and (**F**) 50 µM chloroquine. (**G**) The resulting integrated free energy change per base pair and (**H**) the change in the critical melting force both increase with ligand concentration. Errors represent the standard error from three DNA stretches, where larger than the symbols used.

The free energy of the DNA overstretching transition is calculated as the work done by the force to stretch dsDNA through the transition and to return as one or two melted ssDNA strands. This work can be estimated by integrating the area between the force extension data for dsDNA and ssDNA, as in Figure 5A. Both the integrated area (see Equation (5), Section 4) and the measured transition force increase with increasing chloroquine concentration (Figure 5B–F). The calculated transition free energy change and transition force change as a function of the ligand are presented in Figure 5G,H, respectively. At these concentrations, chloroquine intercalation nearly doubles both the energy and the melting force.

### 2.5. AFM Measures Distinct Binding Modes

**Figure 6:**
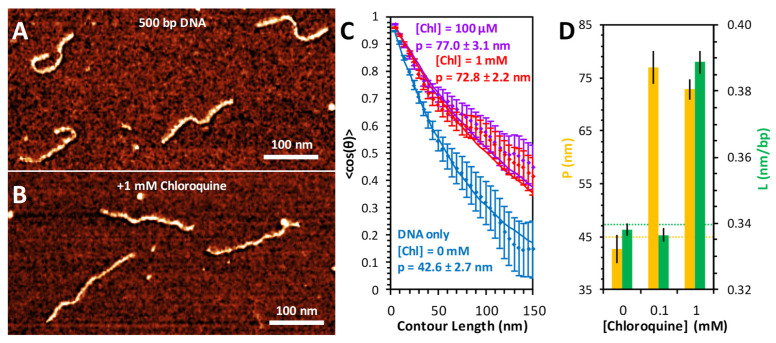
AFM measurement of chloroquine-mediated changes to DNA properties. AFM images of 500 bp dsDNA constructs in the absence (**A**) and presence (**B**) of 1 mM chloroquine. (**C**) DNA molecules are traced to acquire the orientation of the molecule over its entire length. The relative angle change (θ) between every two points separated by contour length ranging from 5 to 150 nm is calculated. The average cosine of this value, averaged over all observed molecules, decays as this length is increased (diamonds with standard error bars). Best fits (solid lines) to the DNA only (blue) and DNA with chloroquine (purple and red) are obtained to determine the persistence length. (**D**) The measured persistence length (yellow) and contour length (green) of DNA–chloroquine complexes. Error bars represent the standard error.

The conformation of DNA and the resulting changes due to chloroquine binding were also measured using AFM imaging (Figure 6). DNA substrates (500 bp) alone appear as semiflexible polymers with variable curvature but consistent length (Figure 6A). Addition of high concentrations of chloroquine visibly straightens and elongates the DNA (Figure 6B). To measure the conformation of the DNA, each individual molecule was traced using segments of uniform length (5 nm) and defined angular orientation. The contour length of each molecule was calculated by summing the total segment length; the persistence length was calculated by the angular correlation of consecutive segments. The average value of the cosine of the change in angle between two segments decays exponentially with their separation length, with the persistence length as a decay constant [20]. Using this method, we found the persistence length of the free DNA to be ~45 nm (Figure 6C). The addition of 100 μM chloroquine nearly doubles this persistence length (Figure 6D). Increasing the chloroquine concentration further, to 1 mM, does not result in further persistence length changes (Figure 6D). This substantial increase happens at chloroquine concentrations about 10-fold lower than required for intercalation, which requires ~1 mM chloroquine under the AFM measurement conditions, with little effect at 0.1 mM (Figure 6D). The presence of Spermidine^3+^ is required for DNA/ligand complex attachment to the mica surface for the AFM measurements. Therefore, concentrations of chloroquine ligand are about 10-fold higher for the AFM measurements compared to the solution measurements, as the chloroquine has to compete with Spermidine^3+^ for DNA binding. The fact that a lower concentration of chloroquine leads to dsDNA rigidification but not elongation suggests that a non-intercalative electrostatic groove binding mode may be responsible for changes in the persistence length [21,22].

### 2.6. Chloroquine Increases dsDNA Melting Temperature

**Figure 7:**
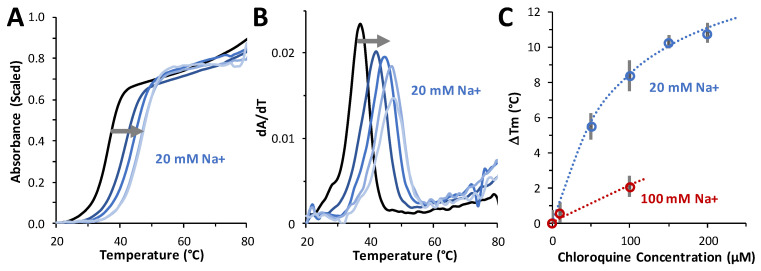
Measuring the effect of chloroquine on DNA stability. (**A**) Thermal melting profiles in 20 mM NaCl of A20:T20 duplex DNA (4 µM dsDNA) in the absence (black) and presence of 50 μM, 100 μM, 150 μM, and 200 μM (from navy to sky blue, following the grey arrow) of chloroquine diphosphate. (**B**) First derivative of melting profiles used to determine *T_m_* at each concentration of chloroquine diphosphate. (**C**) Δ*T_m_* values versus added chloroquine, in 20 mM Na^+^ (blue) and 100 mM Na^+^ (red). Errors (bars) are standard deviations from three independent replicates in 20 mM Na^+^ and estimated from individual profiles in 100 mM Na^+^. Reduced chi-squared fits (dotted lines) determined *K_D_* = 90 ± 10 µM (20 mM Na^+^) and *K_D_* = 650 ± 150 µM (100 mM Na^+^), as described in the text.

Thermal melting experiments were performed to determine the effect of chloroquine on dsDNA duplex stability. For these studies, a 20-bp duplex consisting of dA_20_ annealed to dT_20_ was used. An ~10 °C increase in the *T_m_* was measured in the presence of 200 µM ligand, indicating an increase in the stability of the duplex with ligand bound over DNA alone (Figure 7A–C). The Δ*T_m_* as a function of chloroquine concentrations measured in 20 mM and 100 mM NaCl (in 10 mM HEPES buffer) are plotted in Figure 7C. The maximum chloroquine concentration used in these studies was limited by the fact that chloroquine emits in the same wavelength range as DNA. By fitting these data to a binding isotherm (see Equation (7), Section 4), chloroquine binding at 20 mM NaCl yields a maximum temperature shift Δ*T_m_*(*c_sat_*) of 16 ± 2 °C and a *K_d_* = 90 ± 10 µM. In 100 mM Na^+^, we estimate a *K_d_* of 650 ± 150 µM. Using these two *K_d_* values at two salt concentrations, we estimate a very approximate effective charge of chloroquine intercalating into dsDNA of *Z_eff_* = +0.98 (Equation (7), Section 4). This is a reasonable result for a small molecule with a total charge of +2 (at pH 7.5), as not all of the charges may be affecting the molecule’s ability to displace Na^+^ cations from DNA. This result is consistent with the effective charge of +1 to +2 previously measured for chloroquine [23] and other divalent cationic small-molecule DNA intercalators [24]. As with chloroquine, the intercalators quinacrine and methylene blue stabilized dsDNA with respect to melting by 23 °C and 9 °C, respectively. A similar *T_m_* increase of ~15 °C was also measured for the dsDNA intercalator ethidium [25].

Figure 7C also illustrates the result of fitting to a binding isotherm (Equation (7), Section 4). Chloroquine binding at 20 mM NaCl yields the maximum temperature shift, Δ*T_m_*(*c_sat_*) = 16 ± 2 °C and *K_D_* = 90 ± 10 µM. In 100 mM Na^+^, we estimate the approximate *K_D_* = 650 ± 150 µM in this 5-fold higher salt. Using just these two *K_d_* values at two salts, we can estimate very approximately *Z_eff_* = +0.98 (Equation (7), Section 4). The effective cationic charge of chloroquine intercalating into dsDNA is *Z_eff_* ~ +1. This is a reasonable result for a small molecule with a total charge of +2 (at pH 7.5), as not all of the charges may be affecting the molecule’s ability to displace Na^+^ cations from DNA upon its particular type of binding. This result is consistent with the effective charge of +1 to +2 previously measured for chloroquine (ref) and other divalent cationic small molecule intercalative binding (such as quinacrine and methylene blue) to dsDNA [24]. As with chloroquine, quinacrine and methylene blue stabilized dsDNA with respect to melting by 23 °C and 9 °C, respectively. This *T_m_* increase of ~15 °C was also measured for the widely used dsDNA intercalator ethidium [25].

### 2.7. Chloroquine-B DNA Binding Is Entropically Driven and Endothermic

**Figure 8:**
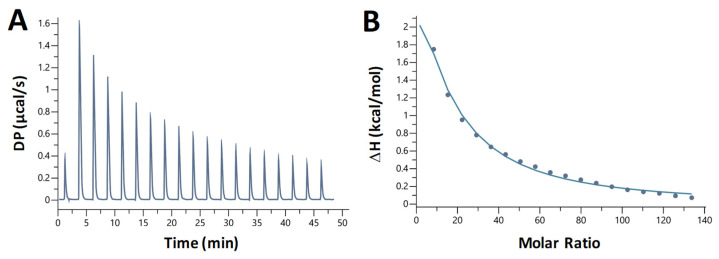
Representative ITC measurement of the interaction of chloroquine with A-T duplex DNA. (**A**) Plot shows the heat of reaction as a function of time. (**B**) Enthalpy change plotted as a function of the chloroquine/DNA molar ratio during the titration.

We also studied the nature of chloroquine—dA_20_:T_20_ duplex DNA binding using isothermal titration calorimetry (ITC). Chloroquine was titrated into a solution containing 10 µM DNA. We observed heat absorption upon chloroquine binding (Figure 8A for an example). The titration was fit to a binding curve (Figure 8B) yielding the following parameters (averaged over three trials): *K_D_* = 360 ± 20 µM, Δ*H* = 21 ± 1 kcal/mol, –*T*Δ*S* = –26 ± 1 kcal/mol, Δ*G* = –4.7 ± 0.3 kcal/mol, and a binding site size of 5, similar to the value obtained from the OT experiments.

Thus, the rather weak chloroquine intercalative binding to dsDNA is driven primarily by the release of entropy and opposed by an almost equally large absorption of heat, i.e., increase in the system’s enthalpy. Entropy-driven dsDNA binding was previously observed for several intercalators [24,26,27]. However, entropy increase is not a universal feature of intercalative binding, as many other dsDNA intercalators were observed to decrease entropy upon binding [28]. Table 1 summarizes the values of *K_D_* obtained in this study from several approaches and in different salts.

## 3. Discussion

Chloroquine was long used as an effective and relatively safe antimalaria and anticancer drug [1]. It is a protonated, weakly basic molecule that exerts its antimalarial and anticancer effects mainly by increasing pH and accumulating in the vacuoles of the parasites [3] and in the internal organelles of cancer cells [29]. The half-maximal effective concentration of chloroquine for malaria and cancer treatment is EC50 ~ 1 μM [30]. Importantly, chloroquine was shown to have a rather narrow therapeutic index, meaning that its cytotoxic concentration (CC50) of ~10–100 μM is close to its EC50 [31]. The initial studies of chloroquine’s anti-COVID-19 activity suggested a similar EC50 of ~1 μM [32] and a CC50 of ~ 100 μM [33]. The mechanism of chloroquine’s anti-COVID-19 (as well as general anti-viral) action in altering the intracellular pH was shown to be similar to its antimalarial activity. However, these studies were largely discontinued, as chloroquine was found to be much less effective in COVID-19 treatment, with an EC50 of ~10 μM, and more cytotoxic, with a CC50 of ~10–100 μM [5,34]. Also, chloroquine was found to have multiple long-lasting side effects in the treatment of COVID-19, likely due to the drug’s long lifetime and accumulation in the organism. The reason for this cytotoxicity, leading to its narrow therapeutic index in COVID-19 treatment, remains unknown.

A recent study [11] has suggested that dsDNA binding by chloroquine may be the reason for its toxicity. Costa et al. used OT stretching experiments to characterize chloroquine–DNA intercalative binding. However, the low forces (<1.5 pN) used in these OT experiments were insufficient to stretch the chloroquine/dsDNA complex to observe its elongation upon chloroquine intercalation in a high-salt buffer; changes in DNA persistence length were observed at low forces and concentrations, consistent with the DNA stiffening we observed using AFM in the present study.

In this work, OT stretching, AFM imaging, chloroquine–DNA melting, and ITC experiments were used to more fully characterize chloroquine–dsDNA binding. Using OT, we find that chloroquine binds dsDNA in a single intercalative mode with a *K_D_* ~ 200 ± 100 μM in physiological salt. We hypothesize that the quinoline moiety is responsible for this intercalation, while the tail does not contribute to this mode (see Figure 1). This is consistent with previous work on pure intercalators such as ethidium [19], indole rings found in cyanine dyes [14,35], and proteins containing intercalating motifs [36]. Saturated chloroquine/dsDNA intercalation occurs every ~5 bp and leads to DNA duplex elongation by 0.29 nm/ligand molecule. This intercalative chloroquine binding is weakly salt-dependent, with an effective charge of *Z_eff_* ~ +1. Our ITC experiments suggest a comparable *K_D_* of ~ 360 μM and a similar binding site size. The intercalative nature of chloroquine–dsDNA binding is reflected in the positive enthalpy Δ*H* = 21 ± 1 kcal/mol coupled with an even larger release of entropy –*T*Δ*S* = –26 ± 1 kcal/mol and a relatively small binding free energy Δ*G* = –4.7 ± 0.3 kcal/mol, suggesting relatively weak intercalation.

The *T_m_* of duplex DNA increased by ~15 °C upon saturated chloroquine intercalation, consistent with the fact that the ligand binds ssDNA much weaker than dsDNA. In addition, in the presence of chloroquine, force-induced stretching results in the transition of B-DNA into S-DNA rather than the energetically more favorable B-DNA strand separation. Because of chloroquine’s ability to stabilize dsDNA, it is likely to slow down dsDNA strand separation, thereby interfering with cellular dsDNA function. Indeed, early studies reported the ability of chloroquine to halt DNA replication and transcription [37], as was also observed for ethidium, although at ~100-fold lower concentrations of the latter. We propose that the intercalative activity of chloroquine observed at ~100 µM may be responsible for the cytotoxicity observed in cells in the same range of drug concentration, and which becomes more pronounced over the time of treatment, likely due to chloroquine accumulation [34].

## 4. Materials and Methods

### 4.1. Samples and Solutions

For single-molecule dsDNA stretching experiments, biotinylated λ-phage dsDNA was tethered between two 3.13 μm streptavidin-coated polystyrene beads (Spherotech, Lake Forest, IL, USA). Biotinylated dsDNA was produced by ligating 5′ and 3′ labels to linearized 48.5 kb λ-phage DNA (Roche, Basel, Switzerland), based on an adapted protocol by Candelli et al. [38]. Similarly, the DNA substrates for the ssDNA stretching experiments were prepared by biotinylating both ends of the linearized baculovirus transfer plasmid pBACgus11 (gift from Borja Ibarra, IMDEA Nanosciencia, Madrid, Spain) [39]. A new stock solution of 10 mM chloroquine diphosphate salt (Sigma, St. Louis, MO, USA) in Ambion^TM^ nuclease-free water (Thermo Fisher Scientific, Waltham, MA, USA) was prepared weekly. The stock solution was further diluted in 100 mM NaCl and 10 mM HEPES at 7.5 pH to different concentrations, as detailed in the figures.

### 4.2. Optical Tweezers

Stretching experiments were performed on a custom-built optical tweezer system [16,39]. Biotinylated λ-phage dsDNA was stretched between two streptavidin beads, with one bead held in the dual-beam laser trap and the other on a glass micropipette tip. A piezoelectric stage moved the micropipette tip while its position provided the controls for DNA extension. The deflection of the trapping laser was used to determine the DNA tension. A custom-built flow cell was connected to four different inlet tubes, allowing for exchange of buffers. First, the streptavidin beads were caught on the tip and the trap. Then, dsDNA was flowed and caught between the beads. DNA was first extended to verify a single tether and confirm the strength of the biotin–streptavidin bond, which should remain at these loading rates up to 100 pN [40,41]. Furthermore, multiple biotins are incorporated on each DNA end; thus, more than one attachment may anchor each end. An excess of chloroquine solution (300–500 μL) was used to exchange the buffer. Standard errors from multiple stretches of dsDNA in buffer and chloroquine solution (≥3) are shown in the figures, unless otherwise noted.

The pBAC dsDNA construct (8.1 kb) was tethered between two streptavidin beads as described above. To melt the dsDNA to ssDNA, 20 μL of 5 M NaOH was flowed over the dsDNA, followed by flushing with 1 mL of buffer. Once the caught strand was confirmed to be single-stranded, chloroquine solution was flowed over the DNA, and multiple stretches on different DNA molecules were recorded.

### 4.3. Models of Polymer Elasticity

The elasticity of dsDNA has been previously characterized by a model that combines entropic flexibility with observed enthalpic elasticity. The extensible worm-like chain (eWLC) model may not be exactly solved, but a high-force limit is known [42]:

(1) $$L(F)=L\cdot \left[1-\frac{1}{2}{\left(\frac{k_{B}T}{F\cdot P}\right)}^{1/2}+\frac{F}{S}\right].$$

Here, *L*(*F*) is the force-dependent measurement of the DNA length, typically the length measured between the beads in force extension data. This length is commonly divided by the number of base pairs, and thus the contour length of any construct is well known to be *L* = 0.34 nm/bp at 30 pN [16]. The persistence length and the stiffness are measures of the entropic and enthalpic elasticity, respectively. Typical values for long DNA in optical tweezers experiments exhibit well-known dependencies on solution conditions, but in the experiments here (10 mM HEPES, pH 7.5, and 100 mM Na^+^), *P* = 45 nm and *S* = 1200 pN.

### 4.4. Deducing Binding Affinity

As an intercalating ligand is introduced, the measured length of DNA increases and continues to increase until ligand binding saturates the double strand, as all binding sites are occupied. This measured change in the length may be expressed as a function of the force-dependent binding site occupancy at a given ligand concentration (Θ(*F*,*c*)):

(2) $$\Theta \left(F,c\right)=\frac{L_{Chl}\left(F,c\right)-L\left(F\right)}{L\left(F\right)}.$$

The force-dependent length in the absence of ligand (*L*(*F*)) is known from Equation (1), while the length induced by ligand binding is measured at varying concentrations of chloroquine *L_chl_*(*F*,*c*). Ligand concentration is also related to the occupancy though a simple isotherm, via the binding site size (*N*) and the equilibrium dissociation constant (*K_D_*):

(3) $$c=K_{D}\cdot \frac{N\Theta }{1-N\Theta }.$$

Thus, fitting the ligand-induced change in the measured length across several forces to Equations (2) and (3) provides both the binding site size and the binding affinity.

Points shown in Figure 3A were taken directly from the average of 3 force–extension curves at every 5th force from 15 pN to 70 pN (for clarity, the figure only shows points for every 10th force). The extension at each selected force for the range of concentrations of chloroquine was fit to the binding isotherms described above. To initiate the fits, *N* was fixed at one while fitting *L_Chl_* and *K_D_*. We obtained *K_D_* and the dsDNA elongation per CHL intercalation event, Δ*x*, by fitting the *K_D_*(*F*) dependence to:

(4) KDF=KDF=0⋅e−FΔxkBT.

Values of *K_D_*(*F* = 0) and Δ*x* were used to find an improved value for *N* according to *N*(*F*) = Δ*x*/(*L_Chl_*(*F*) − *L_DNA_*(*F*)). *N*(*F*) was fixed, and fits of *L_Chl_* and *K_D_* were re-minimized, and *K_D_* and Δ*x* were found again. All errors were propagated from the standard error of three DNA + ligand stretches. These errors were propagated into the chi-squared minimization fits to the binding isotherms. The uncertainties in *K_D_*, Δ*x*, and *n* are determined from χ^2^ + 1 values. 

### 4.5. Calculating the Energy of Overstretching

The change in melting transition free energy was determined as the area in the mechanical cycle between the DNA + Chl stretching curve ($x_{dsDNA-Chlq}(F)$) and the ligand free ssDNA $\left(x_{ssDNA}\left(F\right)\right)$ curve from 0 force to the melting force (*F_m_*) [19]:

(5) $$\Delta G_{DNA-Chl}=\int_{0}^{F_{m}}\left(x_{ssDNA}\left(F\right)-x_{dsDNA-Chl}\left(F\right)\right)\cdot \Delta F.$$

### 4.6. Atomic Force Microscopy

Double-stranded DNA (500 bp) constructs were produced through PCR using a pUC19 plasmid template and then gel purified to ensure uniform length. The DNA was diluted to a concentration of 1 nM in a buffer containing 100 mM NaCl, 10 μM spermidine, and 10 mM HEPES (pH 7.5). Chloroquine was added to the sample at a concentration of 100 nM or 1 mM and allowed to equilibrate for 5 min. The solution (5 μL) was deposited on a freshly cleaved mica surface and then rinsed with deionized water and blown dry after 1 min. The sample was imaged with a MultiMode 8 AFM and Nanoscope V controller (Bruker, Billerica, MA, USA) using the peak force tapping mode with ScanAsyst silicon nitride probes (Bruker, Billerica, MA, USA) and analyzed using Gwyddion software (version 2.55). Custom Matlab (version R2023b, MathWorks, Natick, MA, USA) software was used to trace the DNA molecules following the increased intensity of the DNA backbone relative to the mica surface. Traces were segmented into 5 nm steps with a defined angular orientation. The change in orientation for all segment pairs separated by fixed increments of contour length ranging from 5 nm to 150 nm were averaged to determine persistence length. Total contour length was calculated by summing the total number of segments. Errors in fitted parameters are derived from χ^2^ + 1 values. 

### 4.7. Thermal Melting Studies

Thermal melting experiments were performed in quartz cuvettes. Absorbance at 260 nm was monitored using an Agilent UV–visible spectrophotometer (Cary 3500) equipped with thermoelectrically-controlled cell holders. A 20-bp A:T DNA duplex was formed by heating a dA_20_ oligonucleotide with a dT_20_ oligonucleotide (100 µM each, purchased from Integrated DNA Technologies, Coralville, IA, USA) to 80 °C for 3 min, cooled slowly to room temperature, and placed on ice. The sample was heated in 10 mM HEPES (pH 7.5) and 20 mM or 100 mM NaCl in the absence or presence of varying concentrations of chloroquine diphosphate. The temperature was increased from 15 °C to 95 °C at a heating rate of 1 °C/min. *T_m_* values were determined by taking the first derivative of each melting curve.

Assuming that each chloroquine molecule binds dsDNA independently, we fit the temperature shift vs. chloroquine concentration Δ*T_m_*(*c*) to the simple binding isotherm:

(6) $$\Delta T_{m}\left(c\right)=\Delta T_{m}\left(c_{sat}\right)\cdot \frac{c/K_{D}}{1+c/K_{D}}.$$

Fits to the data determine the temperature shift at saturating concentrations of ligand, Δ*T_m_*(*c_sat_*), and the equilibrium binding affinity (*K_D_*). While full titration was possible in low salt, the melting signal was too noisy at high salt and at high chloroquine concentrations. Thus, fitting of the data determined both Δ*T_m_*(*c_sat_*) and *K_D_* in 20 mM Na^+^. We made the assumption that Δ*T_m_*(*c_sat_*) remained the same in 100 mM Na^+^, allowing determination of *K_D_* at higher salt, although with greater uncertainty.

Measuring *K_D_* in two salt concentrations allowed us to estimate the effective charge of the intercalator (*Z_eff_*):

(7) $$Z_{eff}=-\frac{d\left(\ln\left(K_{D}\right)\right)}{d\left(\ln\left[{\mathrm{Na}}^{+}\right]\right)}.$$

### 4.8. Isothermal Titration Calorimetry

Binding of chloroquine to a 20-bp A:T DNA duplex was measured using a MicroCal PEAQ-ITC (Malvern Panalytical, Malvern, United Kingdom). Chloroquine (7 mM stock in 20 mM NaCl, 10 mM NaPO_4_ (pH 7.5)) was titrated into a sample cell containing 10 μM DNA in a matched buffer in 18 injections of 2 μL at 25 °C. Injections were spaced 150 sec apart to allow the cell to return to equilibrium. The titration experiment took 45 min. Raw data was analyzed using the MicroCal PEAQ-ITC analysis software (version 1.41), and the data was fit using the Levenberg–Marquardt algorithm to determine values for *K_D_*, Δ*H*, and Δ*G*. The number of binding sites (*N*) was constrained to *N* = 4 during fitting.

## Figures and Tables

**Table 1:** Equilibrium dissociation constant (*K_D_*) for chloroquine intercalation into dsDNA determined by thermal melting, ITC, and OT, as described in results.

Na^+^ Concentration (mM)	*K_D_* (µM)	Experimental Method
20	90 ± 10	thermal melting
30	360 ± 20	isothermal titration calorimetry
100	650 ± 150	thermal melting
100	170 ± 90	optical tweezers

## Data Availability

All data generated for this work is available upon request.
